# Unleashing the Genome of *Brassica Rapa*

**DOI:** 10.3389/fpls.2012.00172

**Published:** 2012-07-31

**Authors:** Haibao Tang, Eric Lyons

**Affiliations:** ^1^J. Craig Venter InstituteRockville, MD, USA; ^2^iPlant Collaborative, School of Plant Sciences, University of ArizonaTucson, AZ, USA

**Keywords:** comparative genomics, synteny, CoGe, *Brassica rapa*, syntenic dotplot, *Arabidopsis*, TOC1, conserved non-coding sequences

## Abstract

The completion and release of the *Brassica rapa* genome is of great benefit to researchers of the Brassicas, *Arabidopsis*, and genome evolution. While its lineage is closely related to the model organism *Arabidopsis thaliana*, the Brassicas experienced a whole genome triplication subsequent to their divergence. This event contemporaneously created three copies of its ancestral genome, which had diploidized through the process of homeologous gene loss known as fractionation. By the fractionation of homeologous gene content and genetic regulatory binding sites, *Brassica*’s genome is well placed to use comparative genomic techniques to identify syntenic regions, homeologous gene duplications, and putative regulatory sequences. Here, we use the comparative genomics platform CoGe to perform several different genomic analyses with which to study structural changes of its genome and dynamics of various genetic elements. Starting with whole genome comparisons, the *Brassica* paleohexaploidy is characterized, syntenic regions with *A. thaliana* are identified, and the TOC1 gene in the circadian rhythm pathway from *A. thaliana* is used to find duplicated orthologs in *B. rapa*. These TOC1 genes are further analyzed to identify conserved non-coding sequences that contain cis-acting regulatory elements and promoter sequences previously implicated in circadian rhythmicity. Each “cookbook style” analysis includes a step-by-step walk-through with links to CoGe to quickly reproduce each step of the analytical process.

## Introduction

Cultivars of the *Brassica* genus provide humankind with a wide variety of dietary vegetables and plant oils, and are major contributors to horticultural and agricultural economies worldwide. The *Brassica* crops are frequently used as vegetable cuisine in many cultures where they are recognized as rich sources of dietary fiber, vitamins, and anti-cancer secondary metabolites including glucosinolates and sulforaphane (Hayes et al., 2008). *Brassica* oilseeds, known as “canola oil,” provide about 13% of the world’s supply of edible vegetable oil (Raymer, 2002).

Brassicas display the greatest diversity of leaf and floral architecture, which are manifested among many subspecies within the same species, also known as “morphotypes.” For example, the species *Brassica rapa* includes familiar morphotypes known as Chinese cabbage, bok choy, turnip, canola, etc. *B. oleracea* includes morphotypes such as broccoli, cabbage, cauliflower, Brussels sprouts, and kale. *B. rapa* (A genome), along with its sister species *B. nigra* (B genome) and *B. oleracea* (C genome), make up the “Triangle of U” that describes how the pairwise combinations of these diploid species can hybridize to form allotetraploids, including *B. carinata*, *B. juncea*, and *B. napus* (Nagaharu, 1935). The extreme phenotypic “plasticity” of the diploid and tetraploid *Brassica* species are often compared to the diversity of dogs, both of which are excellent examples for the study of directed artificial selection and the domestication process.

The Brassicas are the closest crop relatives to the model plant species, *Arabidopsis thaliana*, which has a genome size of ∼120 Mb (The Arabidopsis Genome Initiative, 2000; Bennett et al., 2003). The “diploid” *Brassica* genomes are three to five times larger than that of *Arabidopsis*, ranging from 529 Mb for *B. rapa* to 696 Mb for *B. oleracea* (Johnston et al., 2005; Lysak et al., 2009). Earlier studies revealed large chromosomal blocks of conserved synteny and collinearity between *Arabidopsis* and *Brassica* by mapping genetic markers of the *Brassica* genomes onto the *Arabidopsis* reference. These well-conserved regions are often referred to as “Parkin blocks” (Parkin et al., 2003, 2005). The high-resolution whole genome all-against-all comparison between *Arabidopsis* and the recent *B. rapa* genome showed that more than 90% of the sequences from each genome are located in 24 large collinear Parkin blocks (Wang et al., 2011).

**Figure 1:**
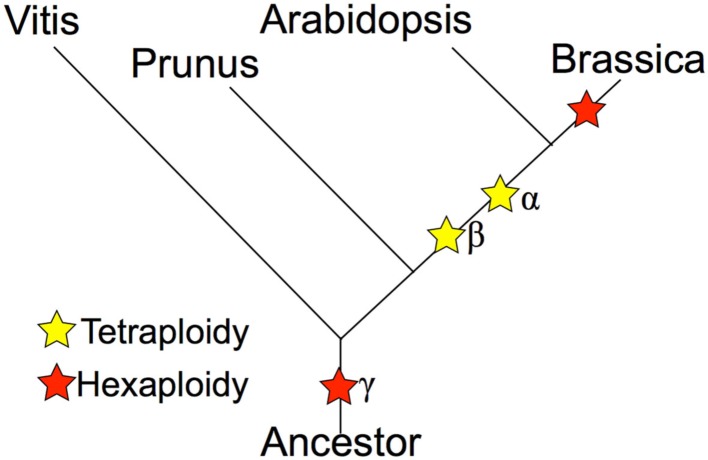
**Phylogeny of *Brassica* and relatives marking the relative placement of lineage divergence and polyploidy events**. Polyploidies are named according to the *Arabidopsis* convention of α (most recent in the linage of *Arabidopsis*), β (second most recent), and γ (eudicot paleohexaploidy).

Whole genome duplications, or polyploidy events, are known to have occurred in the evolutionary history of many plant species (Tang et al., 2008b, 2010; Van de Peer et al., 2009; Jiao et al., 2011; Proost et al., 2011). The model plant *A. thaliana* was already a highly duplicated genome, with three rounds of duplication and triplications (α/β/γ), resulting in at least 12× of the ancestral angiosperm genome (Bowers et al., 2003; Tang et al., 2008a); yet the diploid *Brassica* species has experienced an additional round of genome triplication event on top of these events (Figure 1). The diploid *Brassica* species were first hypothesized to have been triplicated based on comparative mapping (Lagercrantz and Lydiate, 1996; Lagercrantz, 1998; Parkin et al., 2003, 2005), BAC-FISH (Lysak et al., 2005), and BAC sequencing studies (Yang et al., 2006). The genome sequence of *B. rapa* has directly confirmed the genome triplication event with almost complete coverage of the *B. rapa* genome (Wang et al., 2011). The recurring genome duplications and triplication events have created massive genetic redundancy that quickly opens the possibility of sub-functionalization and neo-functionalization for duplicated or triplicated homeologs (Force et al., 1999; Shruti and David, 2005). It is likely that the extreme morphological diversity seen within the various *Brassica* species is due, at least in part, to the genetic redundancy and functional diversification permitted by these genomic events.

Extensive genome-wide comparison between *Arabidopsis* and *Brassica* has revealed unusual patterns of gene loss. The three copies of the genomes within the same nucleus (subgenome) – initially similar in their sizes and gene contents – have since accumulated different amounts of gene losses (or “fractionations”) following the most recent genome triplication event. Of the 24 Parkin blocks of conserved synteny, 20 showed significant deviation from the null “random gene loss” model (Wang et al., 2011; Tang et al., 2012). This striking contrast of gene loss rates among the three distinct subgenomes of *B. rapa* allows each subgenomes to be reconstructed and labeled as subgenomes I, II, III according to the number of gene losses ranging from most (I) to medium (II) to least fractionated (III; Tang et al., 2012). Note that subgenomes I/II/III (Tang et al., 2012) correspond to subgenomes MF2/MF1/LF (Wang et al., 2011), partially due to the fact that the number of genes on subgenomes I and II are very similar and substantially lower than that of subgenome III (Wang et al., 2011). Subgenomes I, II, and III have retained 5966, 7679, and 11536 genes, respectively (ignoring genes that are either unique to *B. rapa* or have transposed; Wang et al., 2011; Tang et al., 2012). Certain classes of genes, particularly subunits of large multimeric protein complexes or regulatory machineries, are retained in higher copy numbers than others (Wang et al., 2011), as predicted by the “Gene Dosage Hypothesis” (Birchler and Veitia, 2010; Schnable et al., 2012b).

By exploiting the close evolutionary relationship between *Arabidopsis* and *Brassica*, researchers have obtained a natural experimental system for understanding the evolution of genome structure following a hexaploidy event. In addition, these lineages are sufficiently diverged to permit the identification of plant conserved non-coding sequences (CNSs; Subramaniam and Freeling, 2012), which may contain cis-regulatory elements. Comparative genomics within the *Brassica* genus and Brassicaceae family will play an increasingly critical role, as many more genome sequences from this family are currently in the making. With a focus on applying these important genomic techniques, this paper uses a set of illustrative questions to walk-through various analyses using the online comparative genomics platform CoGe (Lyons and Freeling, 2008). These questions start with analyzing the entire *Brassica* genome, then dive into specific syntenic regions, and finally analyze promoter sequences of a set of genes to identify putative regulatory sequences. Since all of CoGe’s tools are web-based, the techniques detailed are approachable for anyone with access to a computer and the Internet. However, because many of the analyses have interactive data visualization, using a computer with a large monitor is recommended. All datasets contain in and generated by CoGe are available for download. While *Brassica* is the focus of this paper, the techniques are applicable to any set of genomes, though the interpretations of certain analyses rely on the unique polyploid nature of plants and their relative phylogenetic positions. The examples covered herein include whole genome comparisons within and between *B. rapa* and *A. thaliana* to identify syntenic orthologous and homeologous gene pairs. We will perform in-depth analyses of these syntenic genes sets to reveal the most recent genome triplication event in *Brassica* as well as more ancient polyploidy events in the shared lineages in the crucifer family. We will also provide detailed analyses of a promoter region of a gene involved in the circadian rhythm pathway, TOC1, to identify CNSs and putative cis-regulatory elements.

## Results/Methods/Discussion

### Overview of CoGe

CoGe is publicly available at http://genomevolution.org. This resource contains four major systems: a data engine storing thousands of genomes, a suite of interconnected web-based tools, a wiki documentation system with hundreds of pages on comparative genomics, and a TinyURL resource for storing links to CoGe to regenerate data and analyses. The data in CoGe is constantly growing as new genomes and new versions of existing genomes become available. Currently, there are nearly 20,000 genomes from 15,000 organisms. There are over 20 tools in CoGe; each of these performs one general task, such as searching for genomes, displaying FASTA sequences, querying genomes, comparing genomic regions, etc. These tools are all interlinked with one another so that results generated in one tool may be seamlessly sent to another tool for downstream analyses (Lyons et al., 2008a). Due to the interlinking of these tools, there is no specific workflow or analytical pipeline one must follow. Instead, the questions asked and the discoveries made drive the direction of the analyses.

To learn how to use CoGe, interpret its results, and get background information on comparative genomics, there is an extensive wiki available^1^. Each tool is linked to specific documentation in the wiki, along with links to over 50 written and video tutorials, as well as to FAQs and information about where to get more help.

Most analyses in CoGe return a URL along with the results that can be used to regenerate or share the analysis at any point in the future. It is important to note that there are no inherent pre-computed analyses in CoGe. New analyses are performed on-the-fly. However, large analyses may be cached for some time in case those results are revisited, which will likely incur a one-time computation cost. In order to get the computational scalability needed for its analyses, CoGe is part of the Powered by iPlant program^2^, and makes extensive use of iPlant Collaborative’s compute, storage, and cyber infrastructure resources (Goff et al., 2011). Anyone with an iPlant account may use those credentials to log into CoGe in order to share private data with other CoGe users.

Useful links:

- CoGe: http://genomevolution.org
- Forums: http://genomevolution.org/r/4t7m
- Tutorials: http://genomevolution.org/r/4a3
- New to CoGe: http://genomevolution.org/r/4sr7
- General news: http://genomevolution.org/r/4sr6
- How to get an account: http://genomevolution.org/r/4sr8
- How to add a private genome: http://genomevolution.org/r/4sr9
- CoGe contact list: http://genomevolution.org/r/4tal

### Characterizing the *Brassica* hexaploid

#### Self–self comparisons

The phylogeny in Figure 1 shows that the *Brassica* lineage contains a recent whole genome triplication event. This event has effectively caused the 2*n* ancestor to become a 6*n*. Over evolutionary time, such polyploidy events are followed by the diploidization process, whereby the gene content of a genome is reduced (Wolfe, 2001). The primary mechanism of post-polyploid gene loss is known as fractionation and is thought to occur through deletions by intra-strand recombination events (Woodhouse et al., 2010; Tang et al., 2012). While many duplicated genes are removed by this process, some homeologous genes are retained in multiple copies (Thomas et al., 2006). The retention of these gene pairs provides a strong evolutionary signal of polyploidy events and detection of them permits the identification of duplicated genomic regions (Tang et al., 2008a). Such genomic regions are derived from the same ancestral genomic region and are syntenic. Synteny, in a genomic context, may refer to genomic regions within the same genome or between genomes of different organisms, and are inferred through the identification of collinear sets of putatively homologous gene pairs. The parsimonious reasoning is that a collinear set of homologous genes arose through sharing a common evolutionary history.

Detecting syntenic genomic regions is the high watermark for determining whether a genome underwent a polyploidy event. If, through intra-genomic comparison, all genomic regions are syntenic to other regions, strong evidence is provided for polyploidy. By characterizing the depth of syntenic coverage across a genome, the nature of the polyploidy may be determined. For example, if a genome underwent a tetraploidy event, there would be a 2:2 intra-genomic syntenic mapping where each genomic region is syntenic to itself and one additional genomic region (Tang et al., 2011). Likewise, if a genome underwent a hexaploidy event, there would be a 3:3 intra-genomic mapping (Jaillon et al., 2007).

However, the diploidization process by fractionation can obfuscate the ability to infer synteny through collinear gene order. Over evolutionary time, the more likely any duplicated gene may be lost to fractionation. Fortunately, some gene families are resistant to fractionation, and these can continue to provide a signal to detect syntenic regions. However, concurrent with the diploidization process are additional evolutionary events that can further degrade the collinear signal (Lyons et al., 2008b). These events include gene and genomic region transpositions, chromosomal inversions, chromosomal fissions and fusions, and, most importantly, subsequent polyploidy events. While all of these increase the genomic distance between collinear genes (thus reducing the power to detect syntenic regions), the latter case most effectively reduces the collinear signal by creating an additional duplicate copy of everything in the genome followed by another round of fractionation (Schnable et al., 2012a). This results in syntenic regions of older polyploidy events becoming much more difficult to detect when overlaid by newer ones (Bowers et al., 2003).

**Figure 2:**
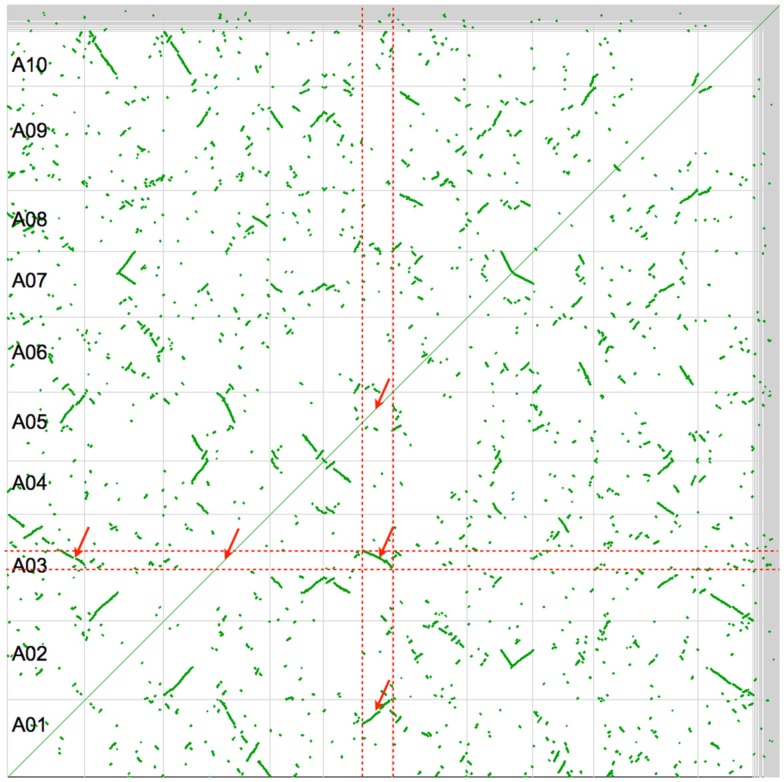
**Syntenic dotplot of self–self *Brassica rapa* comparison**. Horizontal and vertical gray lines separate chromosomes. Green dots are syntenic gene pairs identified through collinearity. Red dashed lines band and red arrows point to large intra-genomic syntenic signal showing a 3:3 syntenic relationship. Results may be regenerated: http://genomevolution.org/r/4srl

Figure 2 shows a self–self syntenic dotplot of the *B. rapa* genome. Only syntenic gene pairs identified through collinearity are drawn on the dotplot (green). When there is a high density of syntenic gene pairs, lines are visualized with varying slopes. The variation in the slopes of syntenic regions is due to biases in fractionation along a genomic region. Syntenic regions cover the entire genome and there are at least two size-classes: large or small. By analyzing a given genomic region by traversing the dotplot vertically or horizontally, it is clear that there are three intra-genomic syntenic regions: the region to itself and to the two larger syntenic regions. The smaller syntenic regions are most likely due to an older whole genome duplication, of which the *Brassica* lineage has had at least three: two tetraploidy events shared with the *Arabidopsis* lineage, and an older hexaploidy shared among nearly all the eudicots (Figure 1).

**Figure 3:**
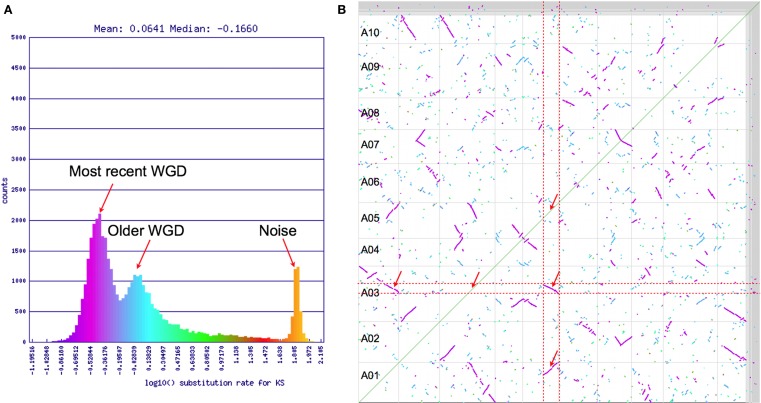
**(A)** Histogram of log 10 transformed *K*s values of syntenic gene pairs identified in Figure 2. **(B)** Syntenic dotplot of self–self *Brassica rapa* comparison with gene pairs colored by their *K*s values shown in (**A)**. Results may be regenerated: http://genomevolution.org/r/4sr4

Synonymous mutation values (*K*s) are often used to determine the relative ages of syntenic gene pairs and the distributions of these values for many pairs of genes may identify unique age classes (Kimura, 1977). If the larger syntenic regions in Figure 2 are indeed from a younger contemporaneous evolutionary event than the smaller regions, they may show a bimodal makeup in their combined *K*s distribution (Blanc and Wolfe, 2004). Figure 3A shows this distribution for log 10 transformed *K*s values for all the syntenic gene pairs identified in Figure 2 as calculated by CODEML (Yang, 2007). Here, there are three conspicuous peaks. The youngest peak, or the peak with the lowest *K*s distance, is on the left. The peak on the far right has a log 10 *K*s value of ∼1.9 (*K*s = 80; 80 synonymous substitutions per site), which is beyond the ability to reliably infer and indicates noise in the analysis. The two left peaks conform to the hypothesis of two recent polyploidy events. The colors from this histogram are overlaid on the dotplot in Figure 3B. This clearly shows that the two size-classes of syntenic regions are each derived from different peaks in the *K*s histogram: larger regions are purple and younger, while smaller regions are cyan and older. Interestingly, there are several very small syntenic regions that are all colored green, which likely are derived from an even older polyploidy event. However, due to their small numbers, their peak in the distribution is not noticeable.

#### CoGe methods

1. Go to CoGe’s homepage. Quick-link: http://genomevolution.org
2. Go to OrganismView. Quick-link: http://genomevolution.org/r/48px
3. Search for “*B. rapa*” using the search box next to “Organism Name.” Quick-link: http://genomevolution.org/r/4srf
4. There may be more than one organism that matches that search term. By selecting different organisms, the page will populate with information about that organism, a list of genomes available for that organism, and information on the selected genome. Search and select for the *B. rapa* genome generated by BGI version 1.1. Quick-link: http://genomevolution.org/r/4srg
5. Under the “Genome information” panel, there is an overview of the genome including its size, number of chromosomes/contigs/scaffolds, the type of sequence (unmasked/masked), links to download the sequence and annotations, and links to various tools in CoGe. Select “SynMap” from the “Links.” This loads SynMap, CoGe’s tool for generating whole genome syntenic dotplots, with this genome selected for both input genomes. Quick-link: http://genomevolution.org/r/4srh
6. Once SynMapis loaded, press “Generate SynMap” to run the analysis. Quick-link: http://genomevolution.org/r/4sri
7. By default, SynMap uses LASTZ (Schwartz et al., 2003; Harris et al., 2010) for the whole genome comparison, the TangTool package (Tang, 2010) for finding tandem duplicates (Tang, 2010; Tang et al., 2011), and DAGChainer (Haas et al., 2004) to identify collinear gene pairs. These options can be adjusted under the “Analysis Options” tab. Based on empirical testing, the fastest algorithm that works well for SynMap is LAST (Kielbasa et al., 2011), which has been recently integrated into SynMap. This algorithm will become the default in the near future.
8. By default, SynMap orders the chromosomes by size along the two axes and uses the nucleotide distance for the axes.
  1. To change this order to be based on the name of the chromosome, select the “Display Options” tab and select “Name” for “Sort Chromosomes by.”
  2. To change the axes distances to genes select “Genes” for “Dotplot axis metric.” Quick-link: http://genomevolution.org/r/4srl
9. SynMap has the option for automatically calculating *K*s values using CODEML for all identified syntenic gene pairs.
  1. To turn this option on, select the “Analysis Options” tab and select “Synonymous (*K*s)” for “CodeML.”
  2. You have the options of also changing the color scheme used, determining whether the values are log 10 transformed, and setting min/max cutoff values. Quick-link: http://genomevolution.org/r/4srm

#### *Brassica* vs. *Arabidopsis* syntenic dotplots

**Figure 4:**
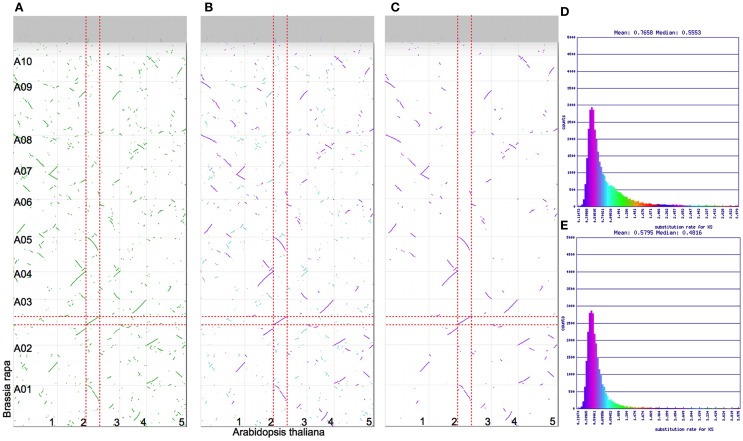
**Syntenic dotplots of *Brassica rapa* (*y* axis) vs. *Arabidopsis thaliana* (*x* axis)**. Vertical and horizontal gray lines separate chromosomes and contigs. Syntenic gene pairs are colored dots on the dotplot. Red dashed lines highlight sets of syntenic regions. **(A)** Syntenic gene pairs colored green. Results may be regenerated: http://genomevolution.org/r/4skv. **(B)** Syntenic gene pairs colored based on their synonymous rate values. Results may be regenerated: http://genomevolution.org/r/4sl1. **(C)** Syntenic gene pairs after screening for a 3:1 (*rapa*:*thaliana*) syntenic depth. These are orthologous gene pairs. Results may be regenerated: http://genomevolution.org/r/4sl5. **(D)** Histogram of synonymous rate values for all syntenic gene-paris. Colors in this histogram are mapped to dotplot shown in **(B)**. **(E)** Histogram of synonymous rate values for syntenic gene pairs screened for a 3:1 syntenic depth. Colors are mapped to dotplot shown in **(C)**.

The *Brassica* hexaploidy event happened after the divergence of its lineage with *Arabidopsis*’ (Figure 1). This means there is a 1:3 mapping of orthologous syntenic regions between *A. thaliana* and *B. rapa*, and a 2:6 syntenic mapping when including their shared most recent tetraploidy event (α; Figure 1). Figure 4A shows a syntenic dotplot between *A. thaliana* and *B. rapa*. There is a strong 1:3 syntenic mapping of large syntenic regions for a given region of *A. thaliana*. As seen in the previous example (Figures 2 and 3), there are many smaller syntenic regions. Applying *K*s value color markups to the dotplot (Figure 4B) highlights the different age classes of the syntenic regions, even though the histogram for these *K*s values does not show a strong bimodal distribution (Figure 4D). The peak corresponding to their shared duplication, the α event (cyan), is much smaller and reflects the degradation of the syntenic signal following the more recent *Brassica* hexaploidy event.

Often when studying genes and genomic regions between organisms, it is useful to differentiate between orthologous syntenic regions and out-paralogous syntenic regions (Koonin, 2005). Figure 4C shows the syntenic dotplot screened for identifying the best syntenic regions giving a 1:3 syntenic depth between *A. thaliana* and *B. rapa* using QUOTA-ALIGN (Tang et al., 2011). This figure retains the *K*s coloration of syntenic gene pairs, and through comparison to Figure 4B, it is clear that nearly all of the retained syntenic regions and corresponding gene pairs are orthologous. When comparing the histograms of the *K*s values from the unscreened dotplot (Figure 4D) to the one screened for a 1:3 syntenic depth (Figure 4E), the tail of the distribution is greatly reduced in the screened histogram. Since all the results of these analyses are available for download (discussed below in “CoGe Methods”), anyone can quickly generate a list of all the orthologous gene sets along with the *K*s values for syntenic gene pairs.

#### CoGe methods

1. Go to CoGe’s homepage. Quick-link: http://genomevolu-tion.org
2. Go to SynMap. Quick-link: http://genomevolution.org/r/4ss0
3. Search for *A. thaliana* by typing “*Arabidopsis*” in the “Name” search box for Organism 1.
  1. There are many matching organisms to this name. Select “*A. thaliana* Col-0 (thale cress; id1)” from the Organism list.
  2. Of several genomes available for *A. thaliana*, select “unmasked (v10, id11022).” Quick-link: http://genomevolution.org/r/4ss1
4. Repeat the search for *B. rapa* for Organism 2 by typing “rapa” in the “Name” search box.
  1. Select organism, “*B. rapa* (id32114),” and the genome, “unmasked (v1.1, id12468).” Quick-link: http://genomevolution.org/r/4ss1
5. Sort the chromosomes by name by selecting the “Display Options” tab and selecting “Name” from “Sort Chromosomes by.”
  1. Run the analysis by pressing the red “Generate SynMap” button. Quick-link: http://genomevolution.org/r/4ss2
6. Turn on the *K*s calculations by selecting the “Analysis Options” tab and selecting “Synonymous (*K*s)” for CodeML.
  1. Rerun the analysis by pressing “Generate SynMap.” Quick-link: http://genomevolution.org/r/4ss3
7. You can adjust the display of the *K*s histogram and colors. To mimic Figure 4B, turn off the “Log 10 Transformation” of *K*s values by clearing the checkbox, selecting “2.2xRainbow” for the color scheme, and selecting a “Max Val” of 4 to exclude the noise peak in the high *K*s range. Quick-link: http://genomevolution.org/r/4sl1
8. To screen for orthologous syntenic regions, select the “Analysis Options” tab and turn on the algorithm by selecting “Quota-Align” from “Syntenic Depth.”
  1. Next, select a syntenic depth of “3” *B. rapa* -to- “1” *A. thaliana*.
  2. The “Overlap Distance” specifies the number of genes by which two syntenic regions may over overlap without either being rejected (Tang et al., 2011). The default value of “40” is usually sufficient. Quick-link: http://genomevolution.org/r/4sl5
9. To download a list of the orthologous syntenic gene pairs from this last analysis, click on “Final syntenic gene set output with GEvo links” available in the “Links and Downloads” section found under the dotplot and *K*s histogram. In this section, you will also, see a link to “Regenerate this analysis” if you wish to return to an analysis in the future. These links were used in the creation of this walk-through.
10. Useful tips:
  - SynMap caches all steps of its analyses. This means that it may take awhile the first time you run a comparison, but the results are returned quickly the next time you run the analysis. If you modify one step in SynMap’s analytical workflow, SynMap uses cached results of the steps leading up to the modified one.
  - SynMap will run faster if CDS (protein coding sequence) is used in the comparison instead of the whole genome sequence.
  - By default, SynMap auto-selects to use CDS if available.
  - When using whole genome sequences, select “masked” sequence, if available.
  - For large genomes (>500 Mb of sequence), there are often a number of repeat sequences caused by transposons. Comparing a large whole genome sequence to itself (especially those containing many young transposons) usually means a very long wait time for the analysis to complete (days to weeks) and uses a large amount of computer resources. Please contact the authors if there is a genome that needs to be masked.

### Identifying syntenic regions of interest

While generating whole genome comparisons is useful for characterizing the evolution between two genomes, many researchers are interested in a particular gene or gene family. The typical method employed for identifying homologs of a particular gene uses BLAST (Altschul et al., 1990) to search genomes of interest for genes of similar sequence. However, such methods are limited in terms of characterizing the evolutionary relationship between genes, and additional analyses are often required to determine whether the genes are related through synteny or other forms of duplication (e.g., tandem duplication, transposition duplication, horizontally transferred). Syntenic dotplots help to some degree as they can be used to find a gene of interest and determine if relatives are present in syntenic regions within the same genome or in a related genome. SynMap in CoGe permits users to zoom in by clicking on a chromosome–chromosome comparison in the dotplot. When the mouse is moved over dots in these zoomed-in comparisons, the crosshairs turn red and information about the gene pair is displayed. Also, clicking on a dot opens CoGe’s tool for high-resolution sequence analysis, GEvo, with genomic sequence surround the selected gene pair preloaded. GEvo will be discussed in the next section. In addition, researchers may download all identified syntenic gene pairs (described above) and can scan through those for their gene of interest using a text editor, spreadsheet, custom program, or command line tools.

CoGe has two additional tools to help identify homologous and/or syntenic genes and regions. One is CoGeBlast, which is CoGe’s interface for BLAST^3^. A detailed explanation of how to use CoGeBlast that is relevant to this discussion is available in Schnable and Lyons (2011). Briefly, CoGeBlast permits researchers to use BLAST to search their sequences of interest against any set of genomes in CoGe; the interactive display of results permits the evaluation of how well the target genome was matched and allows the user to select matched genomic feature (e.g., genes) for downstream analyses (such as GEvo for determining if genes are derived from syntenic regions).

The second tool is SynFind, which identifies all syntenic regions to a given gene in a user-selected set of genomes, regardless of whether the gene is still present in that region. SynFind is powered by an algorithm known as Synteny Score, which is available as part of the Tang Tools (Tang, 2010). The results of SynFind show a table of the matched regions with their synteny scores and whether or not a syntenic gene was identified. There is the option to download all the identified syntenic gene sets anchored on the genome from which the query gene is derived as well as a syntenic depth table. The syntenic depth table is a breakdown of the number genes in the reference genome at a particular syntenic depth. These tables are helpful in characterizing the syntenic relationship between two genomes, especially for contig-level assemblies where it is difficult to visualize large genomic structures using syntenic dotplots. Examples of various syntenic depth tables and their dotplots can be found at http://genomevolution.org/r/4suf. Importantly, at the top of the results page for SynFind is a link to GEvo to permit the analyses of these genomic regions in more detail.

In the following example, orthologs to *A. thaliana*’s TOC1 will be identified in syntenic regions in *B. rapa* using SynFind. TOC1 is part of the circadian rhythm pathway in *A. thaliana* (Strayer et al., 2000) and is one of five members in the PPR protein family (pseudo-response regulators). The PPR family is expressed in succession from morning to night (Matsushika et al., 2000). TOC1 is negatively regulated by the MYB family transcription factors CCA1 (Wang and Tobin, 1998) and LHY (Schaffer et al., 1998), through binding an Evening Element (EE) in its promoter (Alabadí et al., 2001). TOC1, in turn, negatively regulates CCA1/LHY though binding a cis-regulatory element in their promoters called T1ME (Gendron et al., 2012).

#### CoGe methods

1. Go to CoGe’s homepage. Quick-link: http://genomevolution.org
2. Go to SynFind: Quick-link: http://genomevolution.org/r/4suh
3. Search for *Arabidopsis* TOC1 by its TAIR accession by typing “*AT5G61380*” into the “Specify Feature” Name search box.
  1. Press “Search” to run the search.
  2. Select the *A. thaliana* genome that contains “dsgid11022” from the list. Quick-link: http://genomevolution.org/r/4sui
4. Add *B. rapa* to the “genomes to search” list by typing “rapa” into the “Organism Name” search box.
  1. Select the *Brassica* genome with “dsgid12468” and press “+Add.” The genome should appear in the list. Quick-link: http://genomevolution.org/r/4suj
5. Run the analysis by pressing the red “Run SynFind” button. Quick-link: http://genomevolution.org/r/4suj
6. When the results return, you may:
  1. Generate a table of all the syntenic gene sets by clicking the link “Generate master gene set table.”
  2. Generate a syntenic dotplot between two genomes by clicking the “dotplot” link.
  3. Save a link to regenerate the SynFind analysis
  4. Send the identified genomic regions to GEvo for high-resolution analysis of the identified syntenic regions.

### High-resolution analysis of syntenic regions

**Figure 5:**
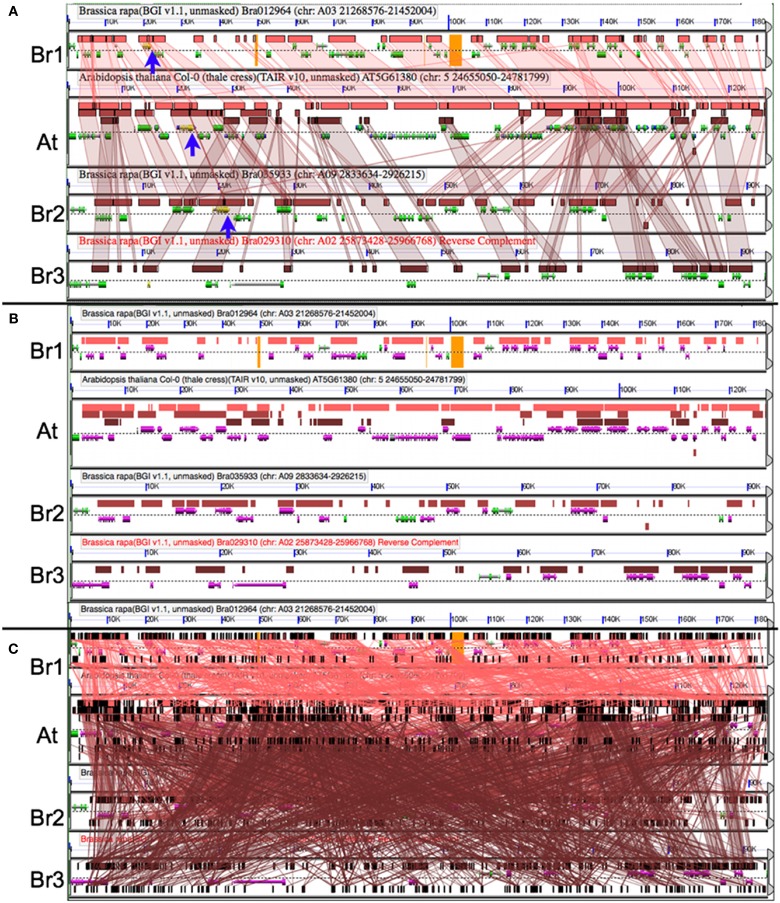
**Comparing orthologous syntenic genomic regions between *Brassica rapa* (Br) and *Arabidopsis thaliana* (At) with GEvo**. Each panel represents a genomic region with the dashed line separating the top and bottom strands of DNA. Orange in the background signifies unsequenced gaps. Gene models are drawn above and below dashed line as composite colored arrows. The At TOC1 gene and its Br orthologs are colored yellow (blue arrows). Regions of sequence similarity are drawn as colored boxes, and may be connected using transparent wedges. **(A)** Syntenic pattern of collinear regions of sequence similarity as identified by LASTZ. Results may be regenerated: http://genomevolution.org/r/4sma. **(B)** Fractionation of Br’s gene content; LASTZ comparison to At’s gene content. Genes covered by a region of sequence similarity are colored purple. Note that At’s gene content is represented among the combined Br regions. Results may be regenerated: http://genomevolution.org/r/4smb. **(C)** Picking the wrong algorithm for the comparison. BLASTN with settings for detecting CNSs used to compare sequences results in too many non-syntenic regions of sequence similarity. Results may be regenerated: http://genomevolution.org/r/4smc

After identifying syntenic regions of interest, it is often useful to analyze those regions in high-resolution. CoGe’s GEvo tool permits the comparison of several genomic regions and provides various ways to modify the analyses and visualization of the results. Figure 5A shows a comparison of the *A. thaliana* genomic region containing TOC1 and the three orthologous syntenic regions in *B. rapa*. In this analysis, all three *Brassica* regions are compared to *Arabidopsis* using LASTZ. Pink-red blocks located above the gene models visualize the regions of sequence similarity. While there is extensive collinear arrangement of similar sequence between these regions, which is strong evidence for these regions being syntenic, note that there are various and different genes missing among the *Brassica* regions when compared to *Arabidopsis*. This is due to the fractionation of gene content. Of the inferred three ancestral copies of TOC1, there are two remaining ancestral copies in *Brassica*. Figure 5B is identical to Figure 5A except that gene models with overlapping regions of sequence similarity are colored purple. This shows that nearly the entire gene content of the *Arabidopsis* region is contained among the *Brassica* regions, even though no one *Brassica* region contains all the gene content of *Arabidopsis*. *Brassica* genes colored green are those that were either lost in *Arabidopsis* or transposed into the region following the divergence of these lineages. Also, note that the sizes of the *Brassica* regions are different (BR1 clearly retains more genes than Br2/3) even though they all have equivalent syntenic coverage of the *Arabidopsis* region. This is due to bias in the fractionation process (Thomas et al., 2006; Freeling, 2009; Schnable et al., 2011; Tang et al., 2012).

An important fact to keep in mind during the comparison of syntenic regions is that different algorithms are better suited for different tasks (Lyons and Freeling, 2008). This generally is due to the fine balance between sensitivity, specificity, and promiscuity of various sequence comparison algorithms. Figure 5C shows the results using BLASTN for comparing the same regions, and its default settings are too sensitive for this type of analysis. As a general rule, use LASTZ (or relatives) for comparing large genomic regions and BLASTN for comparing small regions. However, different algorithms may be better suited for a given problem depending on the intended resolution.

There are two syntenic orthologs of TOC1 identified in Figure 5A in *B. rapa*. It is important to analyze the region with the missing copy in order to determine if the missing gene happened to lie in an unsequenced gap. Such sequences are represented by a string of Ns in the genomic sequence and are colored orange in GEvo. While there are gaps in the *B. rapa* sequence, there are no gaps in the region in which the missing ortholog would be located. Therefore, we can conclude that one of the paleo-orthologs was lost to fractionation.

#### CoGe methods

1. Start with the TOC1 syntenic regions identified with SynFind in the previous analysis. Quick-link: http://genomevolution.org/r/4suj
2. Follow the link to GEvo (top of the results): http://genomevolution.org/r/4sll
3. When GEvo loads, it will have those genomic regions preloaded and will automatically start running the analysis. By default, the query region from SynFind (*Arabidopsis* in this case) will be placed on the top and used as a reference sequence to which all other regions are compared.
4. When the results are returned, click on a region of sequence similarity to connect it with its partner region. For information on how to use the GEvo’s interactive results viewer, see http://genomevolution.org/r/4sz5 (Pedersen et al., 2011).
5. To modify the extent of genomic region analyzed, drag the slider bars located at the end of the genomic regions to zoom in on a region, and either specify an exact amount of sequence up and downstream of the anchor gene, or modify all up and down regions by the same amount.
  1. Expand the analysis by typing “150,000” in the box labeled “Apply distance to all CoGe submissions” and rerun the analysis by pressing the red “Run GEvo Analysis” button. Quick-link: http://genomevolution.org/r/4sz6
6. Use the slider bars to adjust the regions so that only syntenic regions are compared and rerun the analysis. Quick-link:http://genomevolution.org/r/4sma
7. To change the display order of the sequences, drag the sequence submission boxes around relative to one another.
8. To color genes that are overlapped by regions of sequence similarity, select the “Results Visualization Options” tab and turn on the option “Color features overlapped by HSPs” found in the second column. Quick-link: http://genomevolution.org/r/4smb
9. To change the sequence comparison algorithm, select the “Algorithm” tab, and select an algorithm from the “Alignment Algorithm” drop-down menu. Available algorithms are BLASTN (Altschul et al., 1990), LASTZ (Schwartz et al., 2003), CHAOS (Brudno et al., 2004), GenomeThreader (Gremme et al., 2005), LAGAN (Brudno et al., 2003), TBLASTX. Quick-link: http://genomevolution.org/r/4smc

### Regulatory and conserved non-coding sequences

After identifying orthologs to TOC1 in *B. rapa* and confirming their evolutionary history through syntenic analysis, the next step is to identify conserved CNSs in order to identify putative regulatory elements in *Brassica* and to generate hypotheses about their regulatory evolution (Freeling et al., 2007; Subramaniam and Freeling, 2012). Plant CNSs are distinct from animals and have a specific operational definition of two or more similar sequences with an expect value less than or equal to a 15/15 BLASTN exact nucleotide match (Kaplinsky et al., 2002; Inada et al., 2003). Of particular interest, plant CNSs are often detected just above noise when comparing plant sequence (Lyons and Freeling, 2008) and GEvo’s default parameters for BLASTN are set to detect plant CNSs.

**Figure 6:**
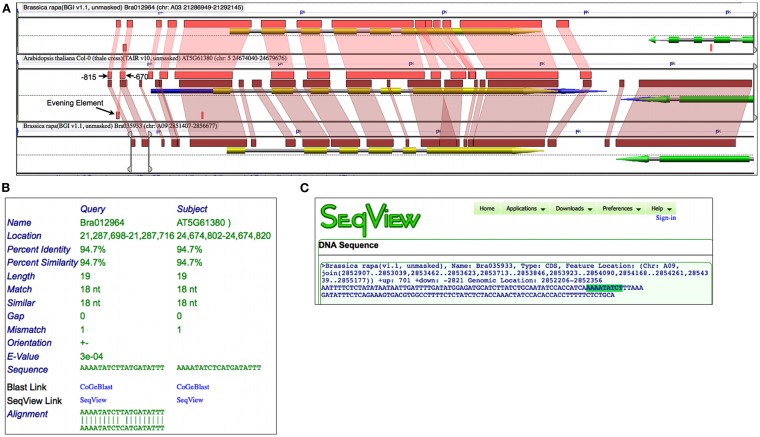
**Conserved non-coding sequence analysis of *Arabidopsis* TOC1 gene and orthologs from *Brassica rapa* using GEvo**. **(A)** Gene models are composite arrows where green or yellow regions represent protein coding sequence, blue is mRNA, and gray is the full extent of the gene. Regions of sequence similar are as in Figure 5 and were identified using BLASTN; such regions are in the opposite orientation if drawn below the dashed line. The bottom *Brassica* region has GEvo’s slider bars adjusted to border the two most 5′ CNSs. Results may be regenerated: http://genomevolution.org/r/4t5e **(B)** HSPView’s report on the BlastN HSP containing Evening Element (AAAATATCT). **(C)** Using SeqView to visualize the sequence encompassing the two most 5′ CNSs in Bra035933 with the Evening Element highlighted. Sequence can be obtained: http://genomevolution.org/r/4t6t

Prior experimental work identified a DNA binding sequence, dubbed EE, in over 30 circadian rhythm cycling genes whose peak expression was at the end of the day (Harmer et al., 2000). An EE sequence was subsequently found in the promoter of *Arabidopsis* TOC1 and, while mutations to the EE caused a strong reduction in circadian rhythmicity, the promoter fragment (−834:−620) containing this element was essential (Alabadí et al., 2001). Figure 6A shows a high-resolution analysis of the *Arabidopsis*’ TOC1 and its two *Brassica* orthologs which includes 1500 nt of sequence up and downstream of the genes. By changing to a higher-sensitivity algorithm such as BLASTN set to detect CNSs, smaller regions of sequence similarity are identified. While, as expected, there is extensive sequence conservation across protein coding regions, there are additional regions of sequence similarity in the CNSs. These CNSs are found 5′, 3′, and in the introns of the genes. Such conservation is assumed to be due to purifying selection providing that enough evolutionary time has passed to randomize non-functional sequences (Freeling and Subramaniam, 2009). By comparing these CNSs with the aforementioned experimental work on the regulation of TOC1, the three most 5′ CNSs identified near *Bra012964* match the promoter fragment in *Arabidopsis* determined to be essential for circadian rhythmicity; one of these CNSs contains an EE (Figure 6B).

While a similar set of CNSs were identified with *Bra035933*, a CNS containing the EE was not detected. However, by extracting out the entire sequence bordered by the two most 5′ CNSs in *Bra035933*, an EE is contained therein (Figure 6C). Interestingly, close examination of these sequences shows that all regions contain a slightly degenerate inverted repeat of EE, which may help to ensure the retention of the sequences during binding-site turnover (Dermitzakis and Clark, 2002), or to facilitate in the cooperative binding of two CCA1/LHY proteins (Eulgem et al., 1999). In any case, analysis of CNSs among homologous syntenic gene sets identifies putative regulatory sequences for further experimental functional characterization.

#### CoGe methods

1. While the slider bars may be adjusted from the GEvo analysis shown in the previous example to border the genes of interest, a faster method is to type “1500” in the box next to “Apply distance to all CoGe submissions.”
  1. Remove the genomic region for *Bra029310* (which does not contain a syntenic ortholog of TOC1) by opening the “Sequence Options” for *Bra029310* and selecting “yes” for “Skip Sequence.”
  2. Make sure that BLASTN is selected for the sequence comparison algorithm under the “Algorithm” tab for increased sensitivity, and leave it on its default settings to detect plant CNSs. Quick-link: http://genomevolution.org/r/4t5e
2. Highlight all of the connections between regions of sequence similarity by holding the Shift key and clicking on a colored box. To get information about a particular region of sequence similarity, click on that colored box without holding the Shift key.
  1. In the “GEvo Results Info” information box, you can view a summary for that particular region of sequence similarity. Click the link called “full summary” to open HSPView, which provides detailed information about the region of sequence similarity. Because the results from GEvo analyses are only cached on CoGe’s server for 2 days, providing a quick-link to HSPView is not possible.
3. Extract the sequence upstream of *Bra035933* by dragging the slider bars to the region shown in Figure 6A and clicking “Get Sequence” from its sequence submission box. Quick-link: http://genomevolution.org/r/4t6t
4. Search for the EE by using the “find” option in your web-browser and typing in AAAATATCT.

## Conclusion

While every genome is sacred, it is essential to have the appropriate computational tools to analyze a genome at various scales. Likewise, comparative analyses of a genome to itself and to related species are required in order to understand how a genome and its genetic components have evolved.

The *B. rapa* genome is of outstanding interest for a variety of reasons. Besides being from an agronomically important and morphological diverse clade of plants, its close phylogenetic relationship to the model plant system *A. thaliana* makes its genome extremely valuable. Due to the timing and phylogenetic placement of the *Brassica* hexaploidy event, and the wealth of information and genetic tools available for *A. thaliana*, the *B. rapa*’s genome provides an exceptional natural experimental system. It is sufficiently diverged from *Arabidopsis* to permit the in-depth characterization of its genome structure, gene retention patterns, and conserved CNSs. The example analyses provided above show how to extract a variety of curious patterns and scientific insights from the *Brassica* genome through comparison to *Arabidopsis*.

The next set of genomic resources of benefit to the *Brassica*, *Arabidopsis*, genome evolution, and gene regulation research communities will be extensive functional genomics data for *B. rapa* such as transcriptomes, small RNAs, and DNA methylation patterns. However, to make the most use of such data, they will need to be integrated into comparative genomics platforms such as CoGe. The vision would be to continue these analyses by overlaying and integrating functional data to investigate the regulation, usage, and timing of TOC1 in *Arabidopsis* and its syntenic orthologs in *B. rapa*. This would permit further characterization of the CNSs found between these sequences and ask questions such as: why has *B. rapa* retained two copies of TOC1 and what is their functional relevance? What is the functional consequence of retaining or losing particular CNSs? Is there something special about the truncation of intron 1 in *Bra012964*? Do these genes have overlapping effects on the functioning of the entire circadian pathway, or have they neo/sub-functionalized their regulation? Sequencing genomes and obtaining their functional data is relatively inexpensive, analyzing these data to transform genomic information into knowledge needs to be too.

## Conflict of Interest Statement

The authors declare that the research was conducted in the absence of any commercial or financial relationships that could be construed as a potential conflict of interest.

## Footnotes

^1^http://genomevolution.org/wiki/

^2^http://www.iplantcollaborative.org/ZkX

^3^http://genomevolution.org/r/4stv
